# The Lagoon at Caroline/Millennium Atoll, Republic of Kiribati: Natural History of a Nearly Pristine Ecosystem

**DOI:** 10.1371/journal.pone.0010950

**Published:** 2010-06-03

**Authors:** Katie L. Barott, Jennifer E. Caselle, Elizabeth A. Dinsdale, Alan M. Friedlander, James E. Maragos, David Obura, Forest L. Rohwer, Stuart A. Sandin, Jennifer E. Smith, Brian Zgliczynski

**Affiliations:** 1 Department of Biology, San Diego State University, San Diego, California, United States of America; 2 Marine Science Institute, University of California Santa Barbara, Santa Barbara, California, United States of America; 3 Hawaii Cooperative Fishery Research Unit, Department of Zoology, University of Hawaii, Honolulu, Hawaii, United States of America; 4 Pacific/Remote Islands National Wildlife Refuge Complex, United States Fish and Wildlife Service, Honolulu, Hawaii, United States of America; 5 CORDIO East Africa, Mombasa, Kenya; 6 Scripps Institution of Oceanography, University of California San Diego, La Jolla, California, United States of America; University of Hull, United Kingdom

## Abstract

A series of surveys were carried out to characterize the physical and biological parameters of the Millennium Atoll lagoon during a research expedition in April of 2009. Millennium is a remote coral atoll in the Central Pacific belonging to the Republic of Kiribati, and a member of the Southern Line Islands chain. The atoll is among the few remaining coral reef ecosystems that are relatively pristine. The lagoon is highly enclosed, and was characterized by reticulate patch and line reefs throughout the center of the lagoon as well as perimeter reefs around the rim of the atoll. The depth reached a maximum of 33.3 m in the central region of the lagoon, and averaged between 8.8 and 13.7 m in most of the pools. The deepest areas were found to harbor large platforms of *Favia matthaii*, which presumably provided a base upon which the dominant corals (*Acropora* spp.) grew to form the reticulate reef structure. The benthic algal communities consisted mainly of crustose coralline algae (CCA), microfilamentous turf algae and isolated patches of *Halimeda* spp. and *Caulerpa* spp. Fish species richness in the lagoon was half of that observed on the adjacent fore reef. The lagoon is likely an important nursery habitat for a number of important fisheries species including the blacktip reef shark and Napoleon wrasse, which are heavily exploited elsewhere around the world but were common in the lagoon at Millennium. The lagoon also supports an abundance of giant clams (*Tridacna maxima*). Millennium lagoon provides an excellent reference of a relatively undisturbed coral atoll. As with most coral reefs around the world, the lagoon communities of Millennium may be threatened by climate change and associated warming, acidification and sea level rise, as well as sporadic local resource exploitation which is difficult to monitor and enforce because of the atoll's remote location. While the remote nature of Millennium has allowed it to remain one of the few nearly pristine coral reef ecosystems in the world, it is imperative that this ecosystem receives protection so that it may survive for future generations.

## Introduction

**Figure 1:**
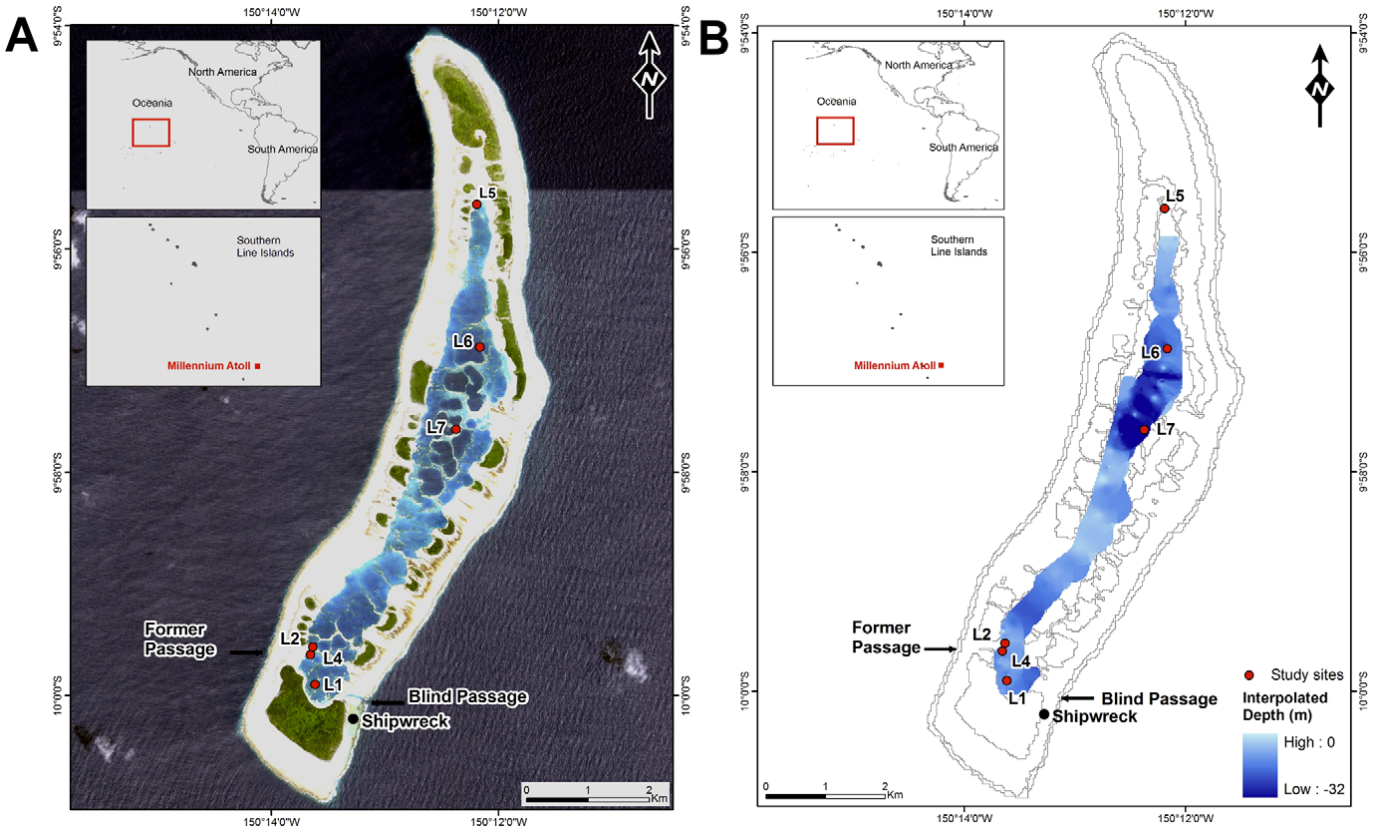
Maps of Millennium lagoon and sites surveyed. A) Satellite map of Millennium indicating the lagoon entrance, shipwreck, former lagoon entrance, and sites surveyed, B) Map of the interpolated depths within the lagoon.

Millennium is among the most remote coral atolls on earth, and is home to some of the most pristine coral reefs. Formerly known as Caroline Atoll and Caroringa, Millennium is a member of the southern group of the Line Islands chain in the equatorial Central Pacific (10°00′S, 150°13.5′W; Figure 1), and part of the Republic of Kiribati. Millennium spans approximately 10 km from north to south and about 2 km east to west. A large central lagoon accounts for the majority of the center of the atoll, measuring about 6 km by 0.5 km, and the shallow perimeter reef supports most of the approximately 39 islets. The three largest islets are Nake Islet, Long Islet, and South Islet, which comprise the majority of the land area. The smaller islets are somewhat ephemeral, varying over time in number, size and shape due to erosion and reshaping following weather events [1], [2]. Rising only 3 m above sea level, the land area of Millennium is under the highest level of threat to sea level rise according to the United Nations [3].

Millennium is distant from human population centers, the closest being 834 km away at Papeete, Tahiti, and has historically been relatively undisturbed by human activity. There are indications of early Polynesian inhabitants on the island, including temple platforms and graves, but no evidence of long-term settlements [1], [2]. Expeditions to Millennium to view a solar eclipse that passed directly over the island took place in 1883 [1], [2]. Guano mining also occurred between 1873 and 1895, and approximately 10,000 tons were reportedly extracted from the atoll, a relatively small amount compared to other islands in the region [1], [2], [4]. Limited copra production and harvesting took place in the early 20^th^ century, but failed due to disease and various animals that destroyed developing trees and nuts (e.g. coconut crabs, rats, and seabirds) [1], [2].

Millennium has been largely uninhabited since the early 1940s, likely due to its remoteness from major population centers, lack of fresh water, and difficulty of anchorage and entrance to the lagoon. The Falconer family settled on the island from 1987–1991, serving as *de facto* caretakers. In 1990, a French businessman Felix Urima began clearing land on the island, harvesting sea turtles and coconut crabs, and fishing around the island in an attempt to start a commercial enterprise. Reports from the three Kepler and Kepler expeditions to Millennium from 1988–1990 [1], [2] led the Republic of Kiribati to cancel Urima's lease [1]. However, as of 1993 the Keplers report that Urima had resumed fishing on the atoll [1]. More recently Millennium was host to the first celebration of the new millennium on January 1, 2000. Realignment of the international dateline in 1995 made the island the first land to greet the dawn of the new millennium, and the atoll was renamed Millennium in honor of this event.

Despite sporadic settlements and brief business ventures on the island, the flora and fauna have recovered well and remain in good condition [1], [2], [4]. Millennium has abundant native flora and fauna (89% indigenous in 1990), including *Pisonia grandis* forests, coconut crabs (*Birgus latro*), and a variety of nesting seabirds, with few invasive organisms (e.g. *Cocos* trees and Polynesian rats [*Rattus exulans*]) [1], [2]. A joint expedition led by the USA and USSR in 1988 included the only known surveys of the marine fauna in the lagoon, but due to time and logistical constraints excluded the majority of the lagoon [5]. Brief observations of the lagoon fauna are also included in the Kepler reports [1], [2]. In April 2009, a series of underwater surveys were conducted throughout the lagoon to characterize the diversity and abundance of fish and benthic organisms during a research expedition to the Southern Line Islands. This is the first extensive survey of the marine natural history of Millennium lagoon. Also included in this report are our observations of indications of human activity in the lagoon, which were present despite its remote location, and the potential significance of this activity on the marine communities surveyed.

## Methods

### Lagoon geography

Research and sample collections were performed under a Scientific Research Permit issued by the Republic of Kiribati for the period of March 24–May 5, 2009. Depth soundings were taken haphazardly around the lagoon using handheld depth sonar with locations recorded using GPS. Measurements were taken throughout all regions of the lagoon except for the most northern lagoon pool. This area could not be reached due to time and logistical constraints. A map of the recorded depths and locations was generated in ArcGIS (Figure 1). Observations of human structures or alterations of the lagoon were also noted.

### Coral diversity and *Tridacna maxima* abundance

Two transects were conducted at each of four sites in the lagoon encompassing 4 regions: south (L1), south-central (L4), north-central (L7), and north (L6) (Figure 1). Transects were conducted on SCUBA at depths between 3–5 m, and covered an area of 25 m×1 m, except transects at site L7, which were 10 m and 14 m, respectively, due to the smaller reefs at that location. Percent cover of benthic organisms was determined using 0.5 m^2^ photoquadrats (n = 6 per transect). Photographs were analyzed by identifying the organisms present under each of 50 randomly generated points in the program PhotoGrid. Benthic organisms were then grouped into the following functional group categories: coral, CCA, turf algae, macroalgae, *Tridacna* (giant clam) and other. Corals whose centers were within the one meter belt transect were identified to genus level and grouped into doubling size classes (i.e. 0–2.5 cm, 2.6–5 cm, 6–10 cm, etc. up to >3.2 m). The density (number per m^2^) of live *Tridacna maxima* clams within each transect was also recorded. A list of coral species was recorded visually at each of the above sites, as well as at one additional site (L5).

### Benthic invertebrates

Holothurian densities were determined visually by counting the number of individuals within a total of 17 transects (50 m×4 m). Transects were conducted haphazardly around the lagoon, including 6 surrounding the patch reefs in the southern region of the lagoon, 10 along the fringing reef of the north-central region, and one around the patch reefs of the north-central lagoon. Depths ranged from less than 1 m along the perimeter reefs to ∼2–5 m deep around the patch reefs.

### Fish abundance and diversity

Belt transect surveys were used to estimate abundances of diurnally active fishes in the reef stratum with the lagoon. Teams of two divers completed paired strip transects (25 m×2 m) at each station. One diver surveyed fishes along the 1 m isobath and the second diver surveyed along the 5 m isobath. All fish observed were identified to lowest recognizable taxon and total length (TL) was estimated to the nearest 5 cm size class. Divers also recorded species observed outside of the transect area to estimate overall species richness at each site. Divers completed 18 belt transect surveys at 9 stations, surveying both patch reef and backreef fringe habitats haphazardly around the lagoon. All surveys were done during the daytime. Thus, species that are primarily cryptic or nocturnal may have been missed.

### Microbial abundance

Water samples were taken at various locations in the lagoon using 5-liter diver-deployed Niskin bottles. Two replicate samples were collected from each site, except the deep lagoon where four replicates were collected. A 10 ml aliquot of the seawater was taken from each Niskin and fixed at a final concentration of 4% paraformaldehyde for 10 minutes in the dark. An aliquot of fixed seawater was then filtered through a 0.02 µm Anodisc filter to trap microbes (Bacteria and Archaea), stained with 1× SYBR Gold for 10 minutes, and mounted onto a glass microscopy slide. SYBR Gold is a general nucleic acid stain that was used to label all microbes, including heterotrophs and autotrophs. All slides were stored at −20°C in the dark until enumeration using epifluorescence microscopy.

## Results and Discussion

### Lagoon geography

The atoll rim is raised slightly above the low tide level, with water exchange into and out of the lagoon occurring over the reef flat and through shallow channels and rills along the reef rim. Water circulation within lagoons is primarily affected by surf, tidal fluctuations, and winds [6], [7]. Based on our visual observations, water flows into the lagoon during high tide and is retained by the lack of deep passes through the perimeter reefs so that at low tide the water level within the lagoon remains higher than that of oceanic water outside the atoll. The tide floods Millennium lagoon from the eastern (windward) side due to wave forcing through and over the reef, creating the east to west flow dynamics within the lagoon. The lagoon appears to drain primarily through the largest channel on the southeast end of the atoll (Figure 1A), which generates an additional north to south flow. The centers of the majority of the pools measured were typically 8.8–13.7 m deep, shoaling along the edges of the pools approaching the reefs (Figure 1B). The deepest pools (>30 m) were in the central region of the lagoon (Figure 1B), much deeper than previous maximum depth estimates of ∼10 m [5].

### Observations of human activity in the lagoon

The largest channel into the lagoon (natural, based upon a 1883 map of the atoll; Figure S1) is located at the southeast limit of the lagoon and adjacent to the south islet, and has been deepened by blasting to allow boats to enter the lagoon and gain shelter from waves (Figure 1A). However, the channel is dead-ended and does not extend fully into the lagoon. A mostly-exposed shipwreck from 1993 currently sits atop of the perimeter reef, just to the south of this pass (Figure 1A). The physical damage to the reef caused by the wreck is still evident, and includes major alterations in the benthic community surrounding the wreck due to iron leaching from the corroding hull. The channel itself is studded with metal stakes rising vertically up from the bottom. Many of these bars reach above the waterline and are easily visible, but a series of rods towards the lagoon side of the passage are submerged and pose a potential hazard to boats. These rods appear to be the remainders of abandoned fish cages, or were placed to discourage boat access to the lagoon. Additional man-made navigation hazards encountered once within the lagoon included lines that have been fixed between some of the patch reefs in the southern region of the lagoon, possibly to discourage unauthorized boat access to the larger lagoon.

At the time of the expedition, there was a small sailboat anchored in the lagoon. The captain had brought the boat onto one of the reef flats and had been living in the lagoon for approximately four months with the intention of staying several more. The captain's activities within the lagoon included harvesting nesting turtles, seabirds, coconut crabs, and giant clams for personal consumption, as well as harvesting lobsters from the reef that were preserved and sold in the nearest ports, primarily in French Polynesia. Most of these (except lobsters) are considered depleted, or threatened by IUCN, and even modest activities such as these have the potential to deplete these otherwise dwindling species and cause long-term injury to the local ecosystem.

### Benthic reef communities

The lagoon consists of at least three major habitat types that vary in structure across both east-west and north-south gradients. First, perimeter reefs and associated back reefs surround each of the islets, and include the continuous reef crest and flats that run between the islets. Second, the interior of the lagoon is dotted with small patch reefs that are often interconnected via deeper coral saddles forming a reticulate reef system including ‘line reef’ structures and coral pillars, pinnacles and bommies. At least two of these line reefs run completely across the lagoon from east to west in the southern portion of the atoll and are exposed at low tide. The third major habitat type within the lagoon is the lagoon floor, which is mostly covered in very fine carbonate sand and hard substrate patches of old coral and relict reef structures.

**Figure 2:**
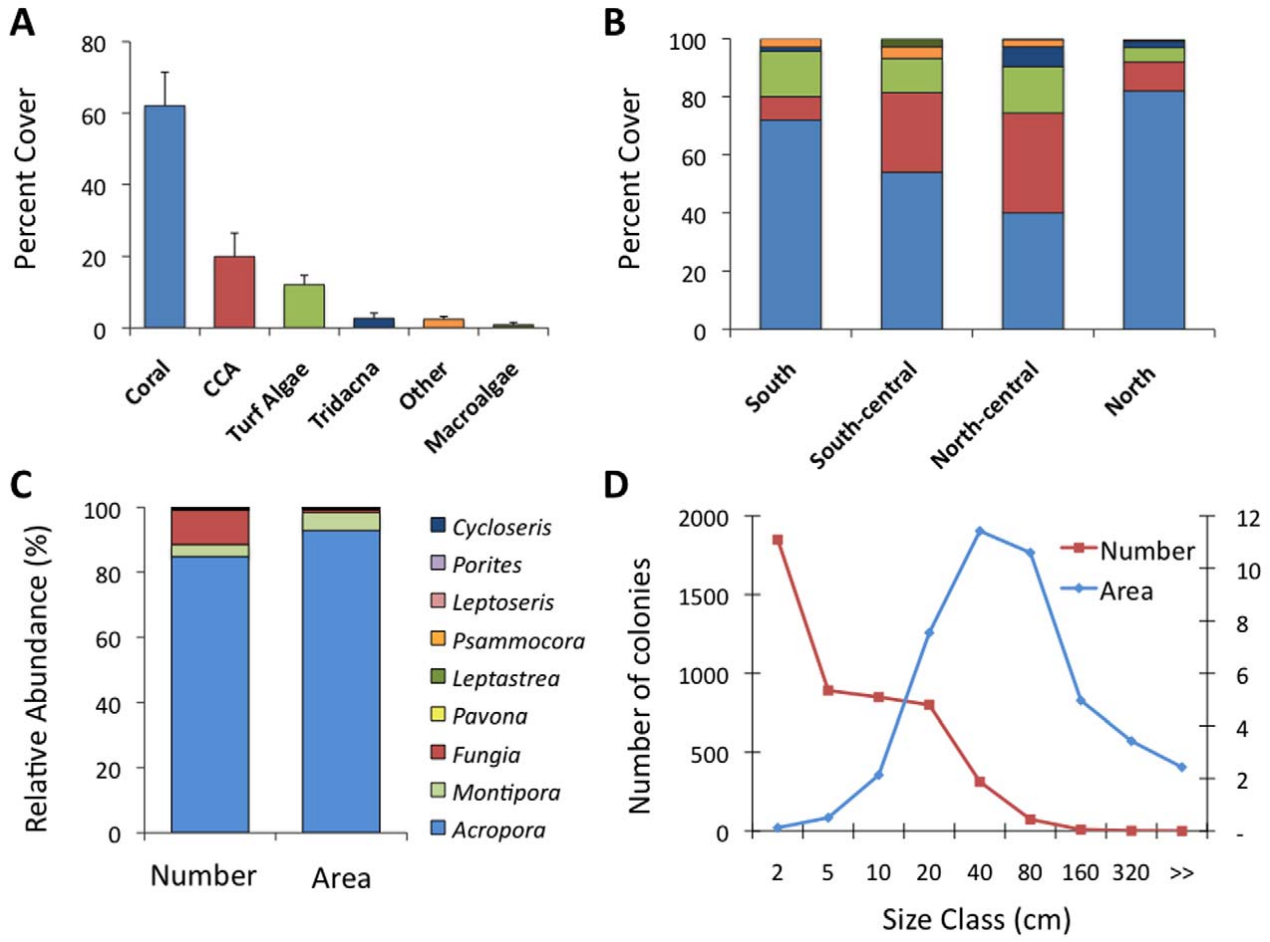
Results of benthic surveys. A) Substrate cover of line reefs in Millenium lagoon (mean±1 standard error). B) Relative percent cover of line reefs by region. C) Relative abundance of coral genera, by number of colonies and percent area cover. D) Size class distributions of corals. The horizontal axis indicates the upper limits of size class.

The line reefs were dominated by hard corals, with an average cover of 62.09 (±9.4, 1 SE; Figure 2A, 3A–B) that varied from near-100% cover to zero. Rubble, generated by breakage of the fragile *Acropora* colonies, was common and often colonized by either CCA or turf algae. CCA were by far the most common algal functional group on the patch reefs (19.9%±6.5) followed by turf algae (12.09%±2.6; Figure 2A). Macroalgal cover was extremely low and mainly consisted of *Halimeda* and *Caulerpa* spp. (mean cover 0.85%±0.6; Figure 2A). Low cover of macroalgae may be due in part to the abundance of herbivores (see below) and heavy competition by corals for bottom space. Coral cover varied by site (Figure 2B), and was greatest in areas of higher water flow within the lagoon (e.g. along the eastern [windward] portions of the lagoon and on pillars and line reefs adjacent to channels and open areas). Coral cover was lowest where water flow was more restricted, such as along the western (leeward) edge of the lagoon and northern edges of the islets as well as in enclosed ‘ponds’ where the line reefs had grown to the surface, restricting lagoon water circulation. The giant clam *T. maxima* was quite common and made up 2.65% of the benthos (±1.5, SE; Figure 2A).

**Figure 3:**
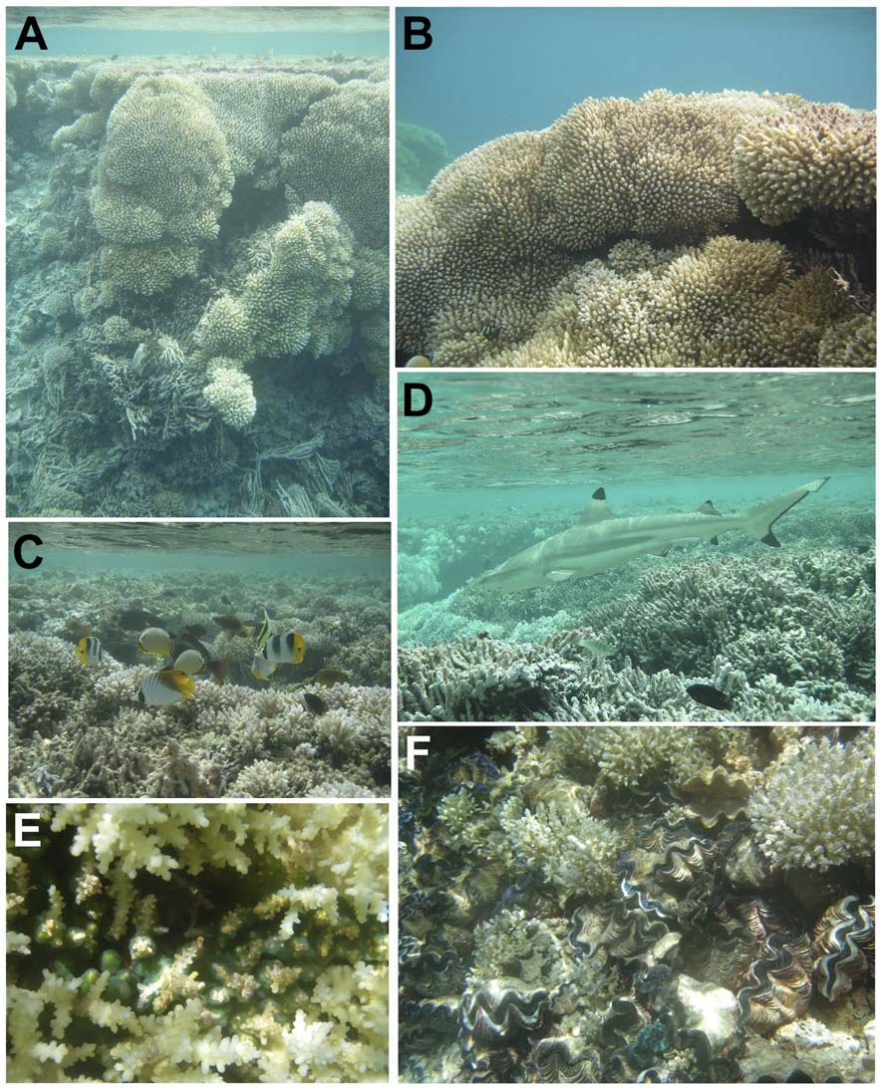
Representative images of common lagoon organisms. A) Patch reef dominated by *Acropora* spp. Tumbling of colonies down the slope along the edge of the reef is evident. B) Example of *Acropora* spp. dominant throughout the lagoon. C) Fish assemblage along the top of a patch reef. D) Blacktip reef shark. E) Ascidians (green) growing in the crevices of Acropora spp. colonies. F) *Tridacna maxima* growing among *Acropora* spp. colonies.

The coral community in the lagoon was dominated by two species of *Acropora* (approx. 90% by area and number of colonies, Figure 2C), followed by *Montipora*, *Fungia*, *Pavona* and *Leptastrea*. A total of 32 species were observed within the lagoon (Table 1). In other environments, the *Acropora* species grow as small cushions, but in the calm waters of the lagoon they grew in very long fingers parallel to one another, with the outer 10–15 cm being alive. The Acroporids dominated the 20–40 cm and 40–80 cm colony size class (Figure 2D). On the most densely covered reefs, colonies >3.2 m were also recorded. We observed a large number of small colonies (<18 per m^2^, Figure 2D) likely due to high levels of both sexual recruitment and asexual fragmentation (visual observations). In the more peripheral parts of the lagoon (north) and pools on the reef flats, *Porites* communities were found, indicative of severe conditions in those areas due to low water circulation and fluctuating temperatures. Other coral genera grew encrusting on the line reefs and pillars, adjacent to or even under the *Acropora* colonies.

**Table 1:** List of 32 stony coral species observed at 5 sites surveyed in Millennium lagoon.

	SITE					
CORAL GENUS/SPECIES	L1	L4	L5	L6	L7	Total # sites observed
***Acropora***						
*Acropora loripes*	1	1	1	0	1	4
*Acropora* sp.	0	0	1	0	1	2
*Acropora subulata*	0	0	0	0	1	1
***Cycloseris***						
*Cycloseris costulata*	0	0	0	1	0	1
*Cycloseris fragilis*	0	0	0	1	1	2
***Fungia***						
*Fungia concinna*	1	0	0	0	1	2
*Fungia granulosa*	0	0	0	1	0	1
***Leptastrea***						
*Leptastrea purpurea*	0	0	0	1	1	2
***Leptoseris***						
*Leptoseris* sp. A [circles]	0	0	0	0	1	1
***Montipora***						
*Montipora caliculata*	0	0	1	0	0	1
*Montipora capitata*	0	0	1	0	0	1
*Montipora dilatata*	0	0	1	0	0	1
*Montipora hoffmeisteri*	1	1	0	1	1	4
*Montipora lobulata*	1	0	0	0	0	1
*Montipora monasteriata*	0	0	1	0	0	1
*Montipora undata*	1	0	0	0	0	1
***Pavona***						
*Pavona chiriquiensis*	0	0	1	0	0	1
*Pavona varians*	0	1	1	0	0	2
*Pavona venosa*	0	0	0	1	1	2
***Pocillopora***						
*Pocillopora damicornis (setchelli)*	0	0	1	0	0	1
*Pocillopora ligulata*	0	0	1	0	0	1
*Pocillopora meandrina*	0	0	1	0	0	1
*Pocillopora* sp. [cockscomb]	0	1	0	0	0	1
***Porites***						
*Porites australiensis*	0	0	1	0	0	1
*Porites lobata*	0	0	1	1	0	2
*Porites lutea*	0	0	1	0	0	1
*Porites* sp. [nodular]	0	0	0	0	1	1
*Porites stephensoni*	0	0	0	0	1	1
*Porites superfusa*	0	0	1	0	1	2
***Psammocora***						
*Psammocora haimeana*	0	1	0	0	0	1
*Psammocora stellata*	0	1	0	0	0	1
***Stylocoeniella***						
*Stylocoeniella armata*	1	1	0	1	1	4
**Total # species observed at each site:**	**6**	**7**	**15**	**8**	**13**	

The line and patch reef structures appear to be a combination of ancient structures and recent coral growth. Live and dead colonies of the massive genera *Leptastrea*, *Goniastrea* and *Favia* could be identified in the framework of the reefs. This was particularly clear at one site in the central lagoon at 30 m depth, where live and dead plates of the coral *Favia matthaii* were abundant, with the latter overgrown by *Acropora* colonies. The local abundance of *F. matthaii* likely reflects isolation of water in the deep pools within the reef, resulting in local seeding, as well as partial mortality, breakage and tumbling of colony fragments, and regrowth. The biggest live *F. matthaii* colony observed was ∼0.5 m in diameter and was unattached on the silty bottom. These observations suggest that massive corals colonize the deep regions of the lagoon, both on hard substrate and loose on the sand, subsequently serving as a settling site for other corals and facilitating the build up of the line reefs. This same progression has been surmised in other lagoons in the Central Pacific with other coral genera that are more widespread and abundant, including *Acropora*, *Leptastrea*, *Goniastrea* and *Fungia* (pers. obs., D. Obura).

The fringing reefs surrounding nearly all of the islets within the lagoon were composed of coral rubble and sand, which was occasionally colonized by cyanobacterial mats (*Schizothrix*). The remaining rubble was either bare limestone or colonized by cyanobacteria, filamentous turf algae and/or crustose coralline algae. These shallow habitats often contain many algal farming damselfishes that aggressively defend their territories; these areas can be easily recognized as dense turf algal patches with one or more damselfish hovering directly above. While the patch and line reefs are largely dominated by *Acropora*, in between many of the live coral colonies the calcified, segmented green alga *Halimeda opuntia* is abundant and perhaps the most common macroalga in the lagoon. Other common macroalgae include two green algae: *Cladophoropsis* sp. and *Caulerpa urvilliana* and the brown encrusting alga *Lobophora variegata*. The top of the patch reefs (∼0.5 m at high tide) were largely colonized by turf algae as they clearly become exposed at low tide, thus limiting growth of corals. Large colonies of cyanobacteria were common on the tops of the patch reefs and can be recognized as a purple-maroon filamentous mass with large oxygen bubbles that are trapped within the colonies. The remainder of these shallow flats was generally colonized by a mixed community of filamentous turf algae appearing as brownish fuzz on the surface of the dead coral skeletons. These turfs can be quite diverse and likely contain upwards of 50 species. Ascidians were also common along bases of the *Acropora* spp. along the tops of the patch reefs (Figure 3E).

**Figure 4:**
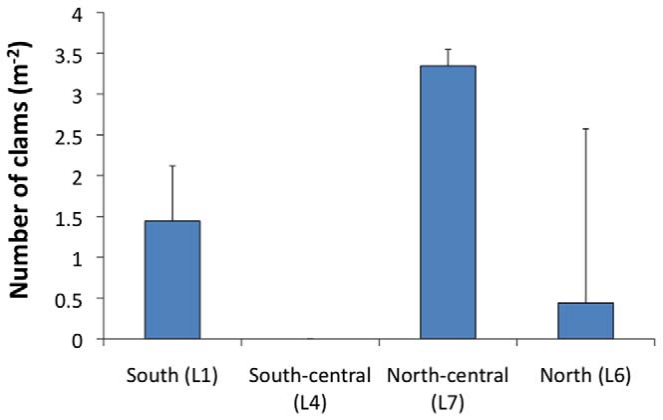
Clam abundance. Number of giant clams, *Tridacna maxima*, at sites throughout the lagoon (mean±1 standard error). Sites are listed from south to north.

*Tridacna maxima* clams were abundant on the line and patch reefs in <10 m water, though highly patchy in distribution (Figure 3F). They were most abundant in areas of high flow and on flat areas just below the lowest low tide height. A high abundance of dead clam shells on raised flats and the tops of line reefs suggests they may be a terminal community in the shallowest depths, suffering mortality to prolonged aerial exposure when water levels are lowered – whether by uplift of the atoll/line reefs, greater opening of the lagoon (therefore more water draining from the lagoon), or by eventual closure of the ‘ponds’. A 1988 expedition to the lagoon found the clams to be highly abundant in the southern region, located in 3–5 m strips along the outward edges of the line reefs and reaching densities up to 80 individuals m^−2^, with an average density of 35 m^−2^ [5]. Only one line reef was surveyed in 1988, but a total 3 of these dense clam beds were observed, all located in the southern region of the lagoon [1], [2], [5]. On this 2009 expedition, over 20 years later, the southern region of the lagoon was littered with dead clamshells and fewer live clams (on average 1.5 m^−2^, Figure 4), although an area of ∼100% clam cover was observed at site L2 in shallow water (Figure 1A). Instead the highest densities of live clams surveyed were observed farther north in the north-central regions of the lagoon (∼3.5 m^−2^, Figure 4). Our surveys were conducted at 3–5 m deep, as opposed to the 1988 surveys that were conducted at 0–1 m deep; however, no clam beds were observed in the shallows that reached the densities observed in 1988.

There are several factors that may have contributed to the decline of the clams in the lagoon. The only navigable entrance is in the southern region of the lagoon, thus making the clams in this area more easily poached than those in the central to northern regions. Kepler and Kepler (1994) reported harvesting of clams for consumption by visiting islanders [1], and current passing seafarers are harvesting the clams (personal observation). While not mutually exclusive, another possibility is that the clams in the south may have died because of infilling or aerial exposure due to uplift. The entrance to the lagoon used during the joint USA/USSR expedition in 1988 [2] on the southwest corner of the lagoon (Figure 1A) had filled in and was impassable in 2009, indicating that there have been changes to the flow and sedimentation in that region.

Holothurians were the most abundant motile macroinvertebrate (0.64 individuals/m^2^) along the shallow edges of the reef flats (∼30–60 cm deep, within 2 m of the reef edge) in the central region of the lagoon. There were very few holothurians observed surrounding central-northern patch reefs, and no holothurians were observed on or surrounding the southern patch reefs. Holothurians were observed in high abundance on the reef flats of the western fringing reefs in the south-central region of the lagoon, although these were not quantified.

### Hypothesis of the lagoon reef structure formation

Reef development is a dynamic process between interactions of water flow, light, the location of hard substrate patches, and extension of reefs by successive periods of coral growth [8], [9]. Both the reticulate line reefs and the coral pillars of Millennium lagoon rising up from the lagoon floor are remnants of historical and current reef growth and erosion processes. As the reefs grow, the pinnacles/hard substrate patches join together in saddles, which influence water flow as the reticulate reef formations grow towards the surface. The relict pillars/hard substrate patches throughout the lagoon indicate that this process has been going on for millennia in a punctuated manner, likely due to sea level changes and disturbance. Millennium lagoon's line reefs generally grow from east to west, perpendicular to the visually predominant current flow from north to south. By orientating across this flow, the reefs are presumably building a wall of mouths, enabling planktivorous organisms (e.g. fish, corals, clams, etc.) to consume plankton entering over the reef [10]–[13], and as a consequence of their dense growth likely deplete the food available downstream (i.e. the southern lagoon).

Similar formation of reticulate reefs also occurs in the lagoons of other Pacific coral reef atolls, including lagoons in the Phoenix Islands (Kanton and Orona), and is likely also the case for lagoons in the Northern Line Islands (Kiritimati, Tabuaeran, and Palmyra before it was dredged). In fact, all of the Line Island Atolls (Palmyra, Tabuaeran, Kiritimati, Millennium), the enclosed lake (former lagoon) at Teraina Island, and even Kingman Reef show reticular reefs that subdivide lagoon areas, based upon imagery in Google Earth (2009). An alternative hypothesis is that reticulated reefs form in lagoons of atolls that are not presently subsiding or are emerging slightly, such as the case for Kiritimati (Christmas Atoll) [14].

The most extreme developments of these reticulate reefs in Millennium lagoon are the two east-west line reefs that traverse the entire width of the lagoon. These two grow up to the surface, thus blocking the flow from north to south. Where the water flow is blocked, the corals eventually die because of loss of food and occurrence of extreme events such as a extremely low tides, bleaching, storm/cyclone/sedimentation, etc., and the centers of these ‘ponds’ were found to have high amounts of rubble and dead coral demonstrating this process. Clams interact with this process as well, growing as the corals do (although limited to the top 10 m) and cementing the tops of the line reefs, while likely enduring more aerial exposure than the corals can tolerate. However, once the line reefs establish a barrier, water flow is restricted and likely leads to death of the filter feeders behind the barrier by restricting influx of water and food. Hydrodynamic investigations and uptake measurements by the benthic community would be necessary to test this hypothesis; however during the present study this data was not collected.

### Fish abundance and diversity

Within the lagoon of Millennium Atoll, the fish assemblages were restricted largely to reef and closely adjacent habitats (Figure 3C). Shallow and deeper soft sediment habitats supported limited density and diversity of fishes within the lagoon. As such, survey efforts were focused on describing the reef fish assemblage. In general, this assemblage showed strong taxonomic overlap with the fauna from the fore reef habitats, though with a significant reduction in total richness and notable shifts in relative dominance.

A total of 89 fish species representing 30 families were recorded from the lagoon at Millennium Atoll during the survey period (Table S1). The most representative family was the wrasses (Labridae, 12 spp.) followed by the surgonfishes (Acanthuridae, 10 spp.), butterflyfishes (Chaetodontidae, 9 spp.), dameselfishes (Pomacentridae, 7 spp.), and parrotfishes (Scaridae, 6 spp.). The total number of fish species observed in the lagoon was low relative to the fore reef (207 spp. found along the 10 m isobath, data not shown), but was not unexpected given the limited amount and homogeneity of the lagoon benthic habitats relative to the fore reef.

Although wrasses contributed the highest number of species, damselfishes were the most numerically abundant taxon with 4 of the 5 most abundant fish species being damselfishes. These damselfishes were found predominately within patches of branching stony corals, especially the dominant Acroporid colonies. The whitetail dascyllus (*Dascyllus aruanus*) was the most abundant species, accounting for over 35% of the total number of fishes and with an estimated density of 2.0 individuals m^−2^, followed next by the blue green damselfish (*Chromis viridis*) with 0.85 individuals m^−2^. The sixbar wrasse (*Thalassoma hardwicke*) was the most abundant wrasse species, accounting for over 10% of the total number of fishes (0.64 individuals m^−2^). The whitebar gregory (*Stegastes albifasciatus*) and neon damselfish (*Pomacentrus coelestis*) were also abundant with 0.43 and 0.25 individuals m^−2^, respectively.

Lagoon and back reef habitats at Millennium Atoll appeared to provide important habitats for recently recruited and juvenile fishes. For example, numerous blacktip reef sharks (*Carcharhinus melanopterus*), many of which were estimated to be juveniles (<100 cm TL), were observed inhabiting shallow back reef habitats throughout the lagoon's perimeter (Figure 3D, Table S1). Similarly, the lagoon at Palmyra Atoll is an important habitat for both adult and juvenile blacktips [15], [16]. In addition, many of the larger (>100 cm TL) adult female sharks on the fore reef during concurrent surveys exhibited mating scars and appeared to be pregnant. The shallow habitats and relative paucity of piscivores in the lagoon (Table S1) relative to the outer reefs (data not shown) may make the lagoon habitats critical for survival of newly recruited and juvenile reef fishes, much like similar habitats in other regions of the world [15], [17]–[22]. Another notable sighting within the lagoon included the Napoleon wrasse (*Cheilinus undulatus*), which was found around many of the patch reefs. Both adults (>50 cm TL) and juveniles (<50 cm TL) were observed; however smaller individuals were more common (Table S1). The largest of the wrasses, the Napoleon wrasse is a fisheries target and is heavily exploited throughout the Indo-Pacific [23], [24]. Populations have become increasingly rare throughout its range resulting in the species being listed as endangered by the IUCN (2004) as well as listed in Appendix II of CITES, and is protected on the Great Barrier Reef. Millennium Atoll may represent one of the few remaining sites in the tropical Pacific where Napoleon wrasse populations can flourish. It is imperative that efforts be made to protect the unique resources and habitats found in the lagoon of Millennium Atoll.

### Planktonic microorganisms

**Figure 5:**
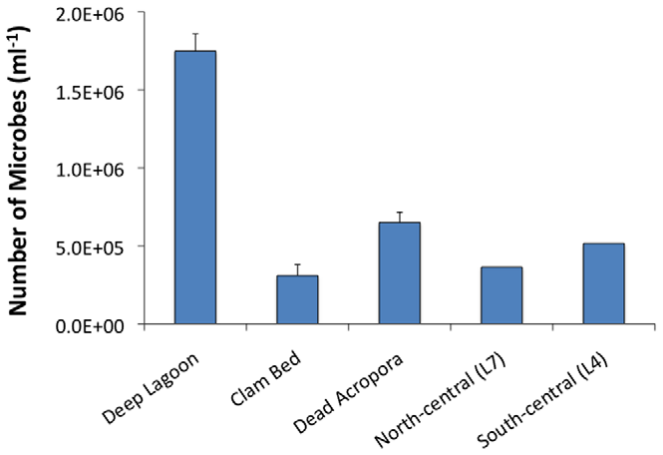
Abundance of microbes within the lagoon. The sites included a deep pool in the central lagoon (n = 4), directly above a clam bed (n = 2), directly above a dead *Acropora* stand (n = 2), south-central lagoon L4 (n = 1), and north-central lagoon L7 (n = 1). Error bars depict standard error.

Microbial abundance (Bacteria and Archaea) in the surface waters of the lagoon at all sites sampled ranged from 3.1–6.5×10^5^ ml^−1^ (Figure 5). The lowest abundance was found directly above the clam gardens (3.1×10^5^ ml^−1^), while water from the deepest area of the lagoon (∼30 m) had the highest microbial abundance (1.8×10^6^ ml^−1^), approximately 400% higher than the surface waters (Figure 5). The higher abundance of microbes in deep areas of the lagoon is likely due to the absence of filter feeders at these depths, although the hydrodynamics and amount of mixing within the lagoon is currently unknown and likely has an affect on microbial and plankton uptake by the benthos and therefore the abundance of these organism in the lagoon [25]. This is the first time that the microbial abundance from Millennium lagoon has been quantified, and provides a valuable reference for microbial abundance in this relatively pristine lagoon.

### Conclusions

This is the first comprehensive survey of the lagoon at Millennium Atoll, which contains some of the few remaining coral reefs that are relatively unaltered by human activity. The lagoon of the atoll is home to a variety of unique organisms that are threatened in many areas of the world. Reef-building corals were the dominant organisms in the lagoon covering greater than 60% of the benthos, a value that is much higher than the regional Pacific-wide average of 22.1% [26] but similar to other uninhabited reefs in the Central Pacific (∼44–56%, [27]). Other reef builders such as CCA were the second most abundant organism with fleshy turf and macroalgae representing a minor component on the benthos, again similar to other uninhabited reefs in the region [27]. The benthic invertebrate community included a variety of filter feeders, including gardens of *Tridacna maxima* in the south to central regions of the lagoon, as well as holothurians along the reef flats and ascidians along the tops of the patch reefs. The clarity of the lagoon water observed may be due in part because of the filtering of these organisms [28]. Fish richness and abundance in the lagoon were generally low, and included occasional visitors from the oceanic reefs. It is likely that Millennium lagoon, like other coral lagoons, serves as an important nursery habitat for many fish species [15], [17]–[22], including important and heavily exploited species such as the blacktip reef shark (*Carcharhinus melanopterus*) and the endangered Napoleon wrasse (*Cheilinus undulatus*) which were observed here.

Protection of Millennium's coral reefs should be a priority for the Republic of Kiritbati as these habitats are not only unique but are some of the world's least impacted reef systems. The relatively undisturbed nature of the lagoon also serves a baseline with which to compare other lagoons in the Pacific Ocean, providing a reference for restoration, management or mitigation of disturbed lagoons. The lagoon of Palmyra Atoll, for example, has been heavily altered by dredging [29]. Restoration efforts can be guided with the goal of achieving similar hydrological dynamics, water quality and coral cover currently found on Millennium. There are several immediate areas for concern regarding conservation of Millennium lagoon. First, if a channel were created to allow for passage of larger vessels into the lagoon, more water would likely drain from the lagoon and the water level of the lagoon would decrease, exposing the corals within the lagoon at low tide. Circulation dynamics within the lagoon would also be altered, and there would potentially be a much higher oceanic influence (e.g. nutrients, temperature, plankton, etc.) and more tidal exchange at depth in the lagoon. Although the effects are unpredictable and complex, these changes would almost certainly alter the biological dynamics within the lagoon. For example, the abundant clam beds found here are typically found in enclosed lagoons around the Central Pacific [30], and opening the lagoon would likely have unexpected effects on the clam population in particular.

A second conservation concern is that there is little to no monitoring or enforcement in the area around Millennium, even though foreign fishing vessels have previously been reported to fish the atoll and temporary residents such as the one encountered during this study are exploiting the island's resources. Valuable resource species such as clams, sharks, Napoleon wrasse, sea turtles, and lobster are fairly abundant at Millennium but have been seriously overexploited elsewhere around the world. These organisms are sensitive to human activities and need to be protected. For example, the clams have been harvested from Millennium in the past [1], and their life histories and easy collection make them vulnerable to overexploitation [31], [32]. Monitoring and enforcement of fishing in the area is crucial, since even a modest amount of extraction of these resources could dramatically alter the lagoon ecosystem, and would likely require a long time to recover due to likely limited repopulation from elsewhere (i.e. the Allee effect [33]).

Lastly, the atoll is likely to experience changes due to the effects of global climate change, such as sea level rise [3], [34], rising sea surface temperatures (SST) and ocean acidification [35]. Widespread bleaching has affected other remote reefs in the Central Pacific, indicating that Millennium may also be subject to this threat. For example, the most sensitive regions of the lagoon of Kanton in the Phoenix Islands experienced near 100% coral mortality due to bleaching caused by abnormally high SST in 2002–3 [36]. At present other reefs in the Phoenix (Baker and Howland Islands) and Line Islands (Palmyra Atoll) are experiencing mass coral bleaching at this time (February 2010, pers. obs. K. Barott, J. Maragos, and G. Williams), and there may be additional bleaching at other Line Islands and atolls to be visited in April 2010 (Jarvis, Kingman). This phenomenon indicates that the remote location of Millennium does not exempt it from the pressure of global climate changes.

## Supporting Information

Figure S1 1883 Map of Millennium Atoll (then Caroline Atoll) from the U.S. Coast and Geodetic Survey Report in 1888.
(5.89 MB TIF)

Table S1 List of fish species observed in the lagoon at Millennium Atoll during the survey period April 16th–24th, 2009 showing the measure of abundance, where: DO = Dominant, AB = Abundant, CO = Common, OC = Occasional, and RA = Rare.
(0.11 MB DOC)
