# Supplementary figures and images for: The Lagoon at Caroline/Millennium Atoll, Republic of Kiribati: Natural History of a Nearly Pristine Ecosystem
Source: PLoS One. 2010 Jun 3;5(6):e10950. doi: 10.1371/journal.pone.0010950 (PMC2880600; doi:10.1371/journal.pone.0010950)

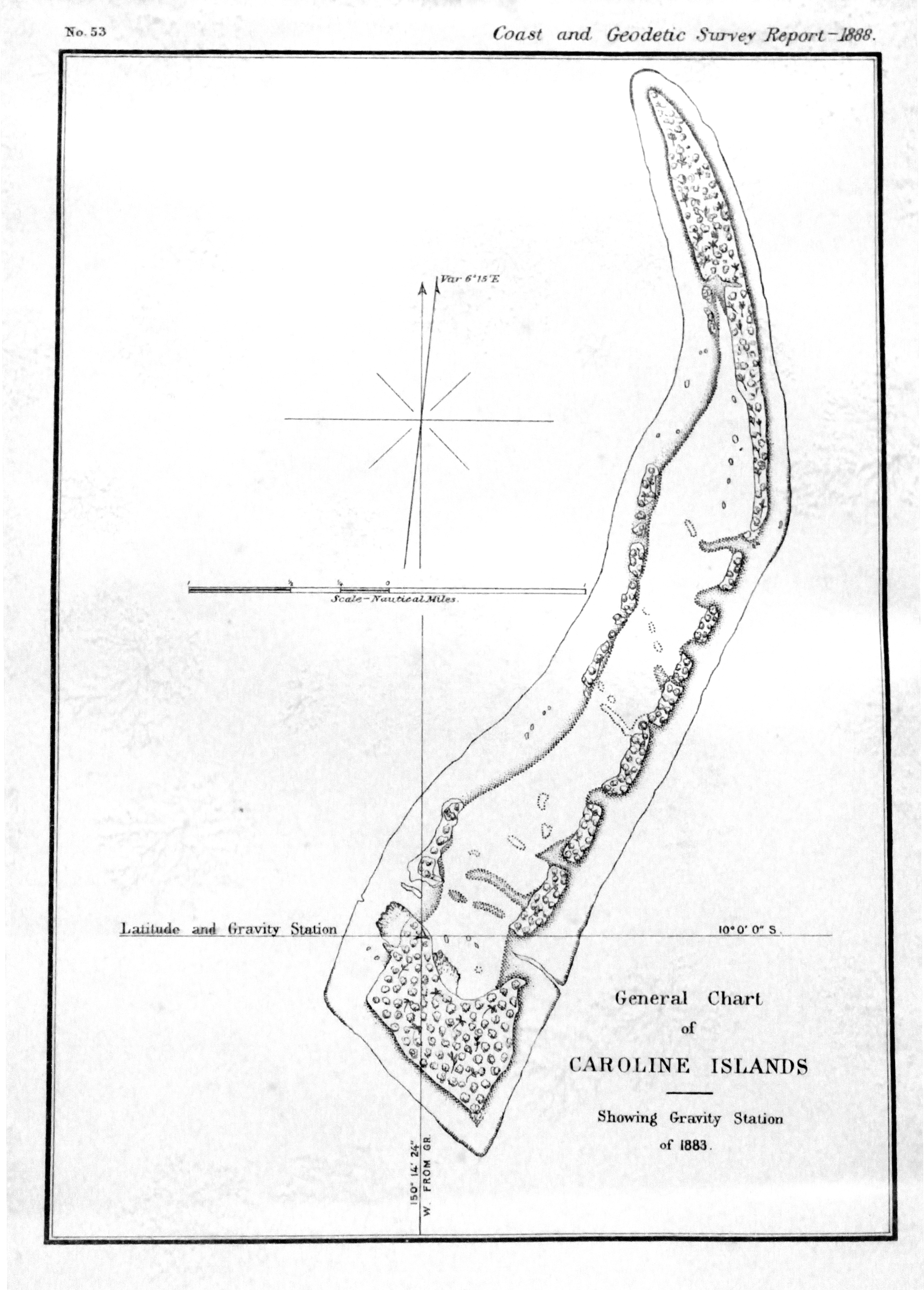

Supplement: Figure S1 — 1883 Map of Millennium Atoll (then Caroline Atoll) from the U.S. Coast and Geodetic Survey Report in 1888. (5.89 MB TIF) [file pone.0010950.s001.tif]
