# Supplementary material for: The Lagoon at Caroline/Millennium Atoll, Republic of Kiribati: Natural History of a Nearly Pristine Ecosystem
Source: PLoS One. 2010 Jun 3;5(6):e10950. doi: 10.1371/journal.pone.0010950 (PMC2880600; doi:10.1371/journal.pone.0010950)
Supplement: Table S1 — List of fish species observed in the lagoon at Millennium Atoll during the survey period April 16th–24th, 2009 showing the measure of abundance, where: DO = Dominant, AB = Abundant, CO = Common, OC = Occasional, and RA = Rare. (0.11 MB DOC) [file pone.0010950.s002.doc]

**Table S1**. List of fish species observed in the lagoon at Millennium Atoll during the survey period April 16^th^ – 24^th^, 2009 Showing the measure of abundance, where: DO = Dominant, AB = Abundant, CO = Common, OC = Occasional, and RA = Rare.

| **FISH FAMILY/SPECIES** | **ABUNDANCE** |
| --- | --- |
| **Carcharhinidae** |  |
| *Carcharhinus melanopterus* (Quoy & Gaimard, 1824) | OC* |
| *Triaenodon obesus* (Rüppell, 1837) | RA |
| **Dasyatidae** |  |
| *Himantura fai* (Jordan and Seale, 1906) | RA |
| **Synodontidae** |  |
| *Synodus sp.* | RA |
| **Mugilidae** |  |
| *Ellochelon vaigiensis* (Quoy & Gaimard, 1825) | RA |
| *Mugil cephalus* (Linnaeus, 1758) | RA |
| **Belonidae** |  |
| **Hemiramphidae** |  |
| **Holocentridae** |  |
| *Myripristis violacea* (Bleeker, 1851) | RA |
| *Neoniphon opercularis* (Valenciennes in C & V, 1831) | RA |
| *Neoniphon sammara* (Forsskål, 1775) | RA |
| **Fistularidae** |  |
| *Fistularia commersonii* (Rüppell, 1838) | RA |
| **Serranidae** |  |
| *Cephalopholis argus* (Bloch & Schneider, 1801) | OC |
| *Epinephelus merra* (Bloch, 1793) | OC |
| *Epinephelus polyphekadion* (Bleeker, 1849) | RA |
| **Cirrhitidae** |  |
| *Paracirrhites arcatus* (Cuvier in C & V, 1829) | RA |
| **Kuhliidae** |  |
| *Kuhlia sp.* | RA |
| **Carangidae** |  |
| *Carangoides orthogrammus* (Jordan & Gilbert, 1882) | RA |
| *Caranx melampygus* (Cuvier in C & V, 1833) | OC |
| *Scomberoides lysan* (Forsskål, 1775) | OC |
| *Trachinotus baillonii* (Lacepède, 1801) | RA |
| **Lutjanidae** |  |
| *Lutjanus bohar* (Forsskål, 1775) | OC |
| *Lutjanus fulvus* (Forster, 1801) | OC |
| *Lutjanus kasmira* (Forsskål, 1775) | RA |
| *Lujanus monostigma* (Cuvier in C & V, 1828) | OC |
| **Lethrinidae** |  |
| *Monotaxis grandoculis* (Bleeker, 1854) | OC |
| **Mullidae** |  |
| *Mulloidichthys flavolineatus* (Lacepède, 1801) | CO |
| *Mulloidichthys vanicolensis* (Valenciennes in C & V, 1831) | RA |
| *Parupeneus cyclostomus* (Lacepède, 1801) | OC |
| *Parupeneus insularis* (Randall & Myers, 2002) | RA |
| *Parupeneus multifasciatus* (Quoy & Gaimard, 1825) | CO |
| **Kyphosidae** |  |
| *Kyphosus vaigiensis* (Quoy & Gaimard, 1825) | RA |
| **Chaetodontidae** |  |
| *Chaetodon auriga* (Forsskål, 1775) | CO |
| *Chaetodon ephippium* (Cuvier in C & V, 1831) | CO |
| *Chaetodon lunula* (Lacepède, 1802) | RA |
| *Chaetodon lunulatus* (Quoy & Gaimard, 1825) | AB |
| *Chaetodon ornatissimus* (Cuvier in C & V, 1831) | RA |
| *Chaetodon quadrimaculatus* (Gray, 1831) | RA |
| *Chaetodon semion* (Bleeker, 1855) | CO |
| *Chaetodon ulietensis* (Cuvier in C & V, 1831) | OC |
| *Chaetodon unimaculatus* (Bloch, 1787) | RA |
| **Pomacanthidae** |  |
| *Centropyge flavissima* (Cuvier in C & V, 1831) | RA |
| **Pomacentridae** |  |
| *Abudefduf septemfasciatus* (Cuvier in C & V, 1830) | RA |
| *Chromis viridis* (Cuvier in C & V, 1830) | DO |
| *Dascyllus aruanus* (Linnaeus, 1758) | DO |
| *Plectroglyphidodon dickii* (Liénard, 1839) | RA |
| *Pomacentrus coelestis* (Jordan & Starks, 1901) | AB |
| *Stegastes albifasciatus* (Schlegel & Müller, 1839) | AB |
| *Stegastes aureus* (Fowler, 1927) | OC |
| **Labridae** |  |
| *Cheilinus undulatus* (Rüppell, 1835) | CO** |
| *Epibulus insidiator* (Pallas, 1770) | OC |
| *Gomphosus varius* (Lacepède, 1801) | OC |
| *Halichoeres trimaculatus* (Quoy & Gaimard, 1834) | CO |
| *Labroides bicolor* (Fowler & Bean, 1928) | RA |
| *Labroides dimidiatus* (Valenciennes in C & V, 1839) | OC |
| *Labroides rubrolabiatus* (Randall, 1958) | RA |
| *Pseudocheilinus octotaenia* (Jenkins, 1901) | RA |
| *Pseudocheilinus tetrataenia* (Schultz, 1960) | OC |
| *Thalassoma hardwicke* (Bennett, 1830) | DO |
| *Thalassoma purpureum* (Forsskål, 1775) | OC |
| *Thalassoma quinquevittatum* (Lay & Bennett, 1839) | OC |
| **Scaridae** |  |
| *Chlorurus frontalis* (Valenciennes in C & V, 1840) | RA |
| *Chlorurus microrhinos* (Bleeker, 1854) | RA |
| *Chlorurus sordidus* (Forsskål, 1775) | AB |
| *Hipposcarus longiceps* (Valenciennes in C & V, 1840) | OC |
| *Scarus altipinnis* (Steindachner, 1879) | RA |
| *Scarus frenatus* (Lacepède, 1801) | OC |
| **Blenniidae** |  |
| *Cirripectes sp.* | OC |
| **Gobiidae** |  |
| *Gnatholepis sp.* | RA |
| **Zanclidae** |  |
| *Zanclus cornutus* (Linnaeus, 1758) | RA |
| **Acanthuridae** |  |
| *Acanthurus achilles* (Shaw, 1803) | RA |
| *Acanthurus guttatus* (Forster, 1801) | RA |
| *Acanthurus nigricans* (Linnaeus, 1758) | RA |
| *Acanthurus triostegus* (Linnaeus, 1758) | OC |
| *Ctenochaetus flavicauda* (Fowler, 1938) | RA |
| *Ctenochaetus striatus* (Quoy & Gaimard, 1825) | OC |
| *Zebrasoma veliferum* (Bloch, 1795) | RA |
| *Naso brevirostris* (Cuvier, 1829) | RA |
| *Naso lituratus* (Forster, 1801) | RA |
| *Naso unicornis* (Forsskål, 1775) | OC |
| **Sphyraenidae** |  |
| *Sphyraena barracuda* (Walbaum, 1792) | RA |
| **Balistidae** |  |
| *Balistapus undulatus* (Park, 1797) | OC |
| *Melichthys vidua* (Solander in Richardson, 1845) | RA |
| *Rhinecanthus aculeatus* (Linnaeus, 1758) | OC |
| *Rhinecanthus rectangulus* (Bloch & Schneider, 1801) | RA |
| **Ostracidae** |  |
| *Ostracion meleagris* (Shaw, 1796) | RA |
| **Tetradontidae** |  |
| *Arothron meleagris* (Lacepède, 1798) | RA |
| *Canthigaster solandri* (Richardson, 1845) | RA |
| **Diodontidae** |  |
| *Diodon holocanthus* (Linnaeus, 1758) | RA |

*Blacktip reef sharks (*Carcharhinus melanopterus*) were commonly observed in the lagoon especially along the sand margins and on the reef flats but were not commonly observed and rare during the quantitative belt transect surveys.

**Napoleon wrasses (*Cheilinus undulatus*), especially juveniles, were commonly observed around patch reef habitats in the lagoon however their shy and elusive behavior prevented them from being recorded during quantitative belt transect surveys.
